# Video laryngoscopy versus direct laryngoscopy in achieving successful emergency endotracheal intubations: a systematic review and meta-analysis of randomized controlled trials

**DOI:** 10.1186/s13643-024-02500-9

**Published:** 2024-03-12

**Authors:** Mohammed Alsabri, Omar Ahmed Abdelwahab, Ahmed Bostamy Elsnhory, Rehab Adel Diab, Vaishnavi Sabesan, Muhammad Ayyan, Christopher McClean, Ayman Alhadheri

**Affiliations:** 1 Department of Emergency Medicine, Al-Thawra Modern General Teaching Hospital, Sana’a City, Yemen; 2 Faculty of Medicine, Al-Azhar University, Cairo, Egypt; 3 Government Kilpauk Medical College and Hospital, Chennai, India; 4 King Edward Medical University, Lahore, Pakistan; 5 Virginia Commonwealth University Health System, Richmond, USA; 6 Michigan State University College of Osteopathic Medicine, East Lansing, USA

**Keywords:** Direct laryngoscope, Emergent intubation, Emergent airway, Endotracheal intubation, First-attempt success, Video laryngoscopy

## Abstract

**Background:**

Intubating a patient in an emergent setting presents significant challenges compared to planned intubation in an operating room. This study aims to compare video laryngoscopy versus direct laryngoscopy in achieving successful endotracheal intubation on the first attempt in emergency intubations, irrespective of the clinical setting.

**Methods:**

We systematically searched PubMed, Scopus, Web of Science, and the Cochrane Central Register of Controlled Trials from inception until 27 February 2023. We included only randomized controlled trials that included patients who had undergone emergent endotracheal intubation for any indication, regardless of the clinical setting. We used the Cochrane risk-of-bias assessment tool 2 (ROB2) to assess the included studies. We used the mean difference (MD) and risk ratio (RR), with the corresponding 95% confidence interval (CI), to pool the continuous and dichotomous variables, respectively.

**Results:**

Fourteen studies were included with a total of 2470 patients. The overall analysis favored video laryngoscopy over direct laryngoscopy in first-attempt success rate (*RR* = 1.09, 95% *CI* [1.02, 1.18], *P* = 0.02), first-attempt intubation time (*MD* =  − 6.92, 95% *CI* [− 12.86, − 0.99], *P* = 0.02), intubation difficulty score (*MD* =  − 0.62, 95% *CI* [− 0.86, − 0.37], *P* < 0.001), peri-intubation percentage of glottis opening (*MD* = 24.91, 95% *CI* [11.18, 38.64], *P* < 0.001), upper airway injuries (*RR* = 0.15, 95% *CI* [0.04, 0.56], *P* = 0.005), and esophageal intubation (*RR* = 0.37, 95% *CI* [0.15, 0.94], *P* = 0.04). However, no difference between the two groups was found regarding the overall intubation success rate (*P* > 0.05).

**Conclusion:**

In emergency intubations, video laryngoscopy is preferred to direct laryngoscopy in achieving successful intubation on the first attempt and was associated with a lower incidence of complications.

**Supplementary Information:**

The online version contains supplementary material available at 10.1186/s13643-024-02500-9.

## Introduction

Preserving clear airways and ensuring appropriate breathing are paramount in caring for critically ill patients [1]. Establishing a definitive airway through endotracheal intubation is the optimal technique, especially in emergencies. However, intubating a patient in an emergent situation presents substantial challenges compared to planned intubation in an operating theater [1, 2].

Various studies have reported different first-attempt intubation success rates for emergent intubations. For instance, a retrospective analysis of 1070 pediatric patients aged 3 to 12 undergoing rapid sequence intubation for elective procedures achieved a remarkable first-attempt success rate of 98.3% [1, 2]. In contrast, studies conducted in various countries have shown success rates for emergent intubations ranging from 52 to 78% [3–5]. These disparities can be attributed to factors such as time and resource constraints in emergencies, patients’ unstable conditions, the absence of pre-screening, and the involvement of less experienced medical personnel, including second-year residents [6].

The complexity of emergent intubations is further compounded by the need for a rapidly assembled team to secure the airway while simultaneously conducting resuscitation efforts and delivering patient-centered care [7]. Adverse events (AEs) during intubation in emergency settings are not uncommon [7]. In a prospective observational analysis involving 2964 patients from 29 countries, approximately 45.2% experienced at least one significant clinical event following intubation [8]. Moreover, the likelihood of unfavorable outcomes, such as aspiration, hypotension, or esophageal intubation, increases with the number of intubation attempts. Patients who require multiple attempts face a higher risk of adverse events [7, 8].

Direct laryngoscopy (DL) has traditionally been the standard approach for intubation. However, recent advancements in video laryngoscopy (VL) have introduced new opportunities to enhance intubation outcomes [9]. Numerous studies have explored the efficacy of video laryngoscopy over direct laryngoscopy during emergent endotracheal intubations. Yet, the results have been somewhat inconclusive, and there has been a lack of a comprehensive analysis specifically focusing on emergency intubations [10–12].

To address this gap in the literature, this meta-analysis aims to systematically review existing randomized controlled trials (RCTs) and compare video laryngoscopy with direct laryngoscopy regarding the first-attempt success rate and other relevant outcomes in emergency intubations.

## Methods

We followed the PRISMA statement guidelines when reporting this systematic review and meta-analysis [13]. All steps were done per the Cochrane Handbook of Systematic Reviews and Meta-analysis of Interventions [14].

### Protocol

Based on the PRISMA guidelines, investigators (M. A. and O. A.) created the review protocol and a search strategy. Our research question was developed following the key elements of the PICO framework: participants, interventions, comparison, and outcomes [15]. The protocol was registered in PROSPERO (international prospective register of systematic reviews) 2023 (CRD42023404181) and is included in the Supplementary material.

### Eligibility criteria

Studies were included in our review if they satisfied the following criteria:

- *Population*: Any adult or pediatric patients undergoing emergent endotracheal intubation for any indication
- *Intervention*: Emergent intubation with VL
- *Comparator*: Emergent intubation with DL
- *Outcome*: The primary outcomes were the proportions of overall intubation and first-attempt success rates. The secondary outcomes were the rates of peri-intubation complications (desaturation (hypoxia), upper airway injuries (oropharynx or dental trauma), esophageal intubation, aspiration, cardiac arrest, and SpO2 < 90), the overall intubation time, the first-attempt intubation time, the peri-intubation percentage of glottis opening (POGO) score, the intubation difficulty score (IDS), and the Cormack-Lehane (CL) grading.
- *Study design*: We included only randomized controlled trials (RCTs).

We excluded studies whose data were not reliable for extraction and analysis, clinical trials with historical control groups, clinical trials in intensive care units, operating rooms, or prehospital settings, simulated patients, or populations, studies that were reported as abstracts only or thesis, studies whose complete full texts were not available, and studies that were not published in the English language.

### Information sources and search strategy

We performed a comprehensive search of four electronic databases (PubMed, Scopus, Web of Science, and Cochrane Central Register of Controlled Trials) from inception until 27 February 2023 using the following query: “(Intratracheal Intubation OR Intratracheal Intubations OR Endotracheal Intubation OR airway management OR tracheal tube) AND (Emergency) AND (Task Performance and Analysis OR checklist OR parameter OR Quality Improvement OR apneic oxygenation OR preoxygenation OR laryngoscope OR laryngoscopy OR video laryngoscopy OR GlideScope OR Pentax OR C-MAC OR blade OR McGrath OR X-lite OR Airtraq OR Truview OR CEL-100 OR ‘King vision’ OR Bullard OR Venner OR vividtrac OR copilot VL).” Additionally, we manually searched the references of the included studies, Google Scholar, and Research Gate for additional articles of interest.

### Selection process

EndNote (Clarivate Analytics, PA, USA) was used to remove duplicates. The collected references were screened in two phases: the first step consisted of screening the titles/abstracts of all the articles identified independently by two to determine their relevance to this meta-analysis, and the following phase consisted of screening the full-text versions of the included abstracts for final suitability to meta-analysis.

### Data collection process and data items

Data were extracted to a uniform data extraction sheet. The extracted data included the following: (1) Characteristics of the included studies (study ID, groups (interventions/exposures), country, type of laryngoscope, and the number of participants), (2) characteristics of the population of included studies (age, sex, body mass index, indication for intubation, physician postgraduate year, and potential airway difficulty), (3) risk-of-bias domains, and (4) outcome measures (the proportions of overall intubation and first-attempt success rates, the rates of peri-intubation complications (desaturation (hypoxia), upper airway injuries (oropharynx or dental trauma), esophageal intubation, aspiration, cardiac arrest, and SpO2 < 90), the overall intubation time, the first-attempt intubation time, the peri-intubation POGO score, the IDS, and the CL grading.

### Risk of bias in the individual studies and publication bias across the studies

We used the Cochrane assessment tool 2 (ROB2) for randomized controlled trials [16]. The risk-of-bias assessment included the following domains: bias arising from the randomization process, bias due to deviations from intended interventions, bias due to missing outcome data, bias in the measurement of the outcome, bias in the selection of the reported result, and other bias. The authors’ judgments are categorized as “low risk,” “high risk,” or “some concerns” of bias.

To investigate publication bias across studies, funnel plots were constructed to illustrate the correlation between effect size and standard error. The following two methods were used to assess the evidence of publication bias across studies: fail-safe N, Mazumdar rank correlation test (Kendall’s tau), and regression test for funnel plot asymmetry using Jamovi software (version 1.6 for Windows).

### Statistical analysis

For categorical variables, such as the overall intubation and first-attempt success rates, we used the risk ratio (RR) to estimate the effect size and compare between video laryngoscope and direct laryngoscope groups. For continuous variables, such as the first-attempt intubation time, we used the mean difference (MD) to estimate the effect size to assess the difference in outcome measures between video and direct laryngoscope groups. All effect estimates were pooled with the corresponding 95% confidence interval (CI), and the *P*-value of less than 0.05 was considered a statistical significance.

The chi-square test (Cochrane Q test) was used to assess statistical heterogeneity between studies. The I-squared was then calculated using the chi-square statistic, Cochrane Q, according to the following formula: *I*^2^ = $\left(\frac{{\text{Q}}-{\text{df}}}{{\text{Q}}}\right) \times 100\mathrm{\%}$. We deemed heterogeneity to be significant if the chi-square *P*-value was less than 0.10. I-square values below 50% indicated a high level of heterogeneity.

When there was no heterogeneity, we used the fixed-effect meta-analysis model, and when there was a significant heterogeneity, we used the DerSimonian Laird meta-analysis model [17] employing Review Manager software (version 5.4 for Windows). The funnel plots and publication bias tests were generated by Jamovi (version 1.6 for Windows).

We conducted a certainty assessment using sensitivity analysis (also known as leave-one-out meta-analysis) to measure the reliability of the evidence. For each outcome of the meta-analysis, we performed sensitivity analyses excluding a single study from each scenario to ensure that the overall effect magnitude was independent of any single study.

## Results

### Literature search results

**Fig. 1:**
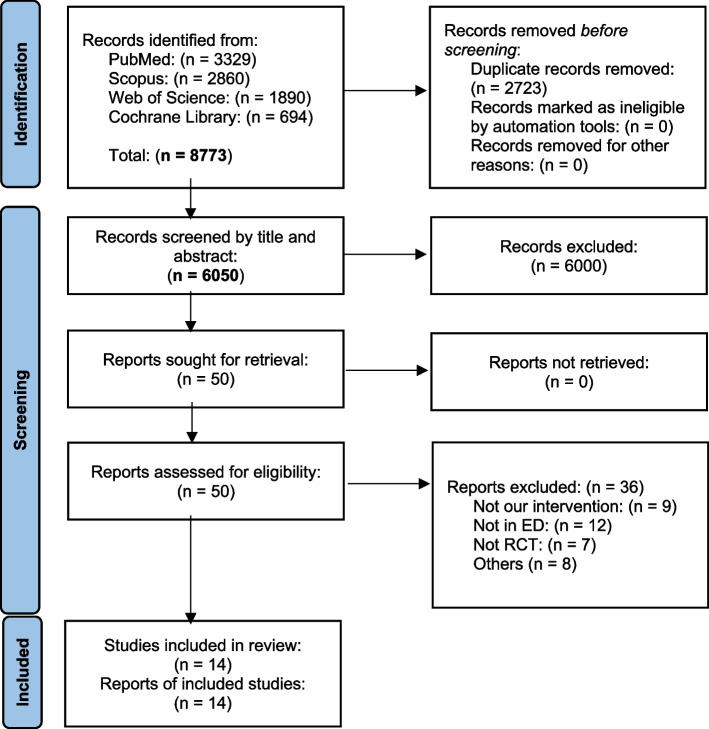
PRISMA flow diagram of studies’ screening and selection

Our literature search process retrieved 8773 records. Following title and abstract screening, 50 articles were eligible for full-text screening. Of them, 14 studies were included in the meta-analysis. The references of the included studies were manually searched, and no further articles were included. The PRISMA flow diagram of the study selection process is shown in (Fig. 1).

### Characteristics of the included studies

**Fig. 2:**
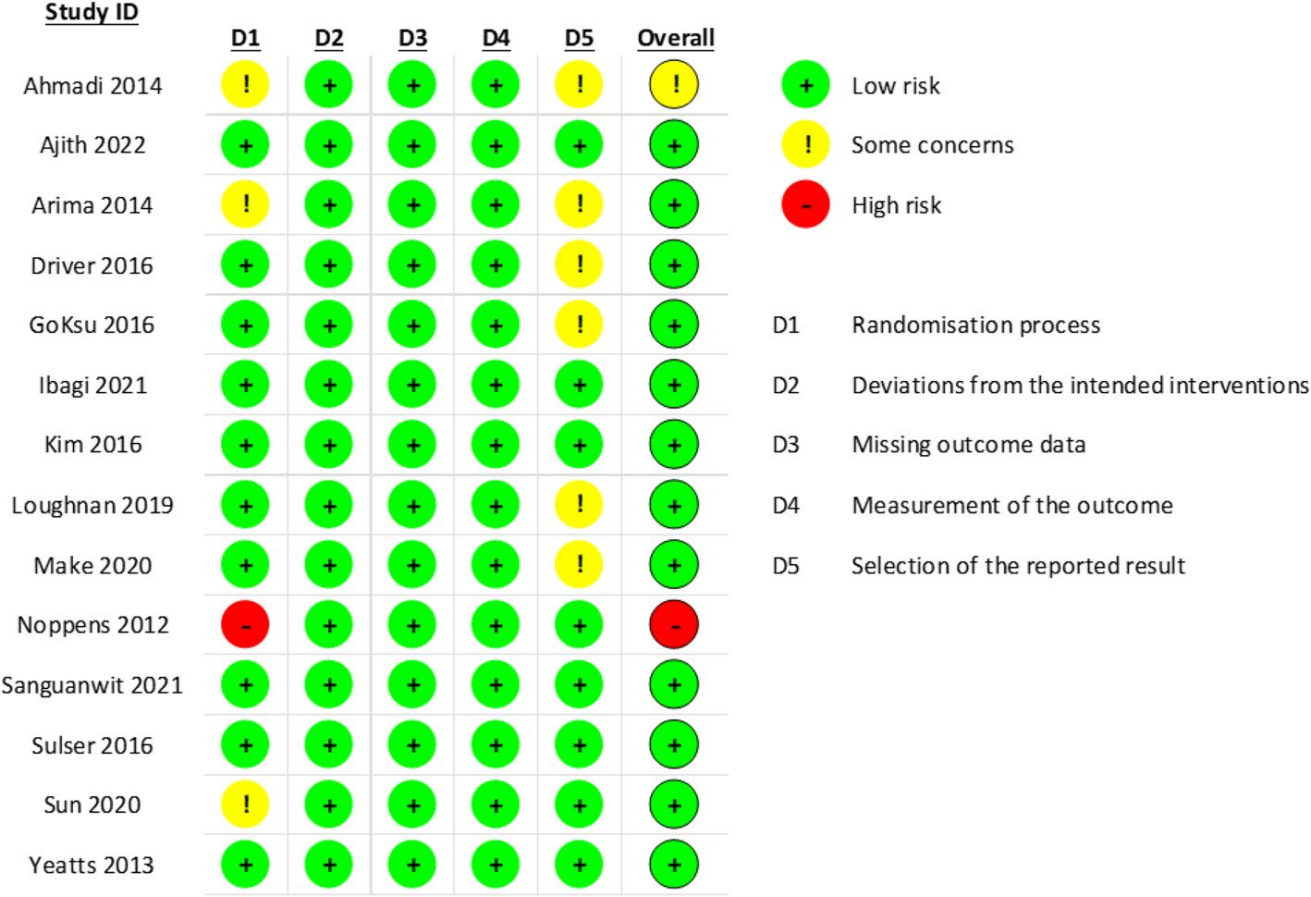
Risk-of-bias assessment of the included studies

Fourteen studies were included in the meta-analysis with a total of 2470 patients. In all studies, patients were assigned to undergo either video or direct laryngoscopy. A summary of the characteristics of the included studies is provided in Tables 1and 2. Overall, the risk of bias was low in most of the included studies (*n* = 12), and only one study ranked to have a high risk of bias, and only one study has some concerns risk of bias, according to the ROB-2 tool (Fig. 2).

**Table 1:** Summary of the studies included in this systematic review and meta-analysis

Study ID	Groups (interventions)	Country	Population	Type of laryngoscope
Ahmadi (2014) [18]	Direct-L	Iran	Patients admitted to ED and need emergency intubation	Macintosh laryngoscope
	Video-L			GlideScope video laryngoscope
Ajith (2022) [19]	Direct-L	India	Critically injured patients requiring emergency intubation	Macintosh laryngoscope
	Video-L			McGrath MAC video laryngoscope
Arima (2014) [20]	Direct-L	Japan	The study included patients who were 18 years or older and required emergency tracheal intubation during daylight hours in the prehospital setting	Macintosh laryngoscope
	Video-L			Airway scope (AWS; Pentax)
Driver (2016) [21]	Direct-L	USA	Participants who had undergone emergent intubation or were at high risk of requiring intubation	C-MAC video laryngoscope with covered screen
	Video-L			C-MAC video laryngoscope
Goksu (2016) [22]	Direct-L	Turkey	Patients over the age of 16, arriving at the ED due to blunt trauma requiring endotracheal intubation to secure the airway	Standard laryngoscope
	Video-L			C-MAC video laryngoscope
Ilbagi (2021) [23]	Direct-L	Iran	Patients with suitability for intubation, age between 18 and 65 years, the absence of anatomical issues in the neck and trachea, and no history of drug addiction	Macintosh laryngoscope
	Video-L			GlideScope video laryngoscope
Kim (2016) [24]	Direct-L	Korea	Adult patients who suffered out-of-hospital or inhospital cardiac arrest and required intubation during cardiopulmonary resuscitation	Standard laryngoscope
	Video-L			GlideScope video laryngoscope
Loughnan (2019) [25]	Direct-L	New Zealand	All adult patients (age > 18 years old) who needed endotracheal intubation upon arrival at the emergency department	Macintosh laryngoscope
	Video-L			McGrath MAC video laryngoscope
Macke (2020) [26]	Direct-L	Germany	Patients needed emergency intubation	Standard laryngoscope
	Video-L			C-MAC video laryngoscope
Noppens (2012) [27]	Direct-L	Germany	Patients in the ICU and need intubation	Macintosh laryngoscope
	Video-L			C-MAC video laryngoscope
Sanguanwit (2021) [28]	Direct-L	Thailand	All adult patients (age > 18 years old) who needed endotracheal intubation upon arrival at the emergency department	Standard laryngoscope
	Video-L			GlideScope video laryngoscope
Sulser (2016) [29]	Direct-L	Switzerland	Patients aged between 18 and 99 years undergoing emergency rapid sequence intubation in the emergency room	Macintosh laryngoscope
	Video-L			C-MAC video laryngoscope
Sun (2020) [30]	Direct-L	China	Patients with respiratory failure, who needed endotracheal intubation upon arrival at the emergency department	Not clearly defined
	Video-L			Not clearly defined
Yeatts (2013) [31]	Direct-L	USA	All patients who required tracheal intubation	Standard laryngoscope
	Video-L			GlideScope video laryngoscope

**Table 2:** Baseline characteristics of the included studies

Study ID	Groups (interventions/exposures)	No. of participants	Age mean (SD)	Sex (male) *n* (%)	BMI mean (SD)	Indication for intubation *n* (%)				
						**Pulmonary**	**Trauma**	**Neurologic**	**Fluid overload, cardiac/renal**	**Other**
Ahmadi (2014) [18]	Direct-L	49	49.1 (12.5)	35 (73)	23.82 (5.21)	-	-	-	-	-
	Video-L	48	52.3 (14.1)	29 (59.2)	22.1 (4.72)	-	-	-	-	-
Ajith (2022) [19]	Direct-L	38	-	-	-	-	-	-	-	-
	Video-L	38	-	-	-	-	-	-	-	-
Arima (2014) [20]	Direct-L	56	74.1 (13)	38 (71.7)	-	-	-	-	-	-
	Video-L	53	74.4 (13.6)	34 (60.7)	-	-	-	-	-	-
Driver (2016) [21]	Direct-L	95	52.6 (17.1)	62 (60)	-	66 (64)	10 (10)	-	4 (4)	13 (13)
	Video-L	103	51.6 (18.8)	63 (66)	-	52 (55)	16 (17)	-	5 (5)	8 (8)
Goksu (2016) [22]	Direct-L	75	39 (19)	70 (93.3)	25.3 (4.53)	14 (19)	33 (44)	4 (5.3)	11 (14.7)	-
	Video-L	75	35 (15.5)	73 (97.3)	24 (3.02)	13 (17.3)	25 (33.3)	5 (6.7)	16 (21.3)	-
Ilbagi (2021) [23]	Direct-L	35	45.7 (11.3)	20 (57.1)	-	-	-	-	-	-
	Video-L	35	45.3 (11.2)	16 (45.7)	-	-	-	-	-	-
Kim (2016) [24]	Direct-L	69	60.5 (18.7)	49 (71.0)	-	-	-	-	-	-
	Video-L	71	61.3 (18.5)	45 (63.4)	-	-	-	-	-	-
Loughnan (2019) [25]	Direct-L	49	52.3 (17.56)	30 (61)	28.3 (5.9)	-	-	-	-	-
	Video-L	51	53.7 (20.6)	25 (49)	28.53 (5.2)	-	-	-	-	-
Macke (2020) [26]	Direct-L	76	Median (Q: Q3) 68 (55:78)	113 (74.3)	-	2 (2.6)	27 (48.7)	5 (6.6)	41 (53.9)	4 (5.2)
	Video-L	76			-	2 (2.6)	24 (31.6)	6 (7.9)	29 (38.2)	2 (2.6)
Noppens (2012) [27]	Direct-L	140	62.9 (15.6)	87 (64.9)	26.9 (7.6)	83 (59.3)	-	8 (5.7)	3 (2.1)	33 (23.6)
	Video-L	134	63.8 (16.7)	86 (64.2)	26.7 (6.4)	86 (64.2)	-	9 (6.7)	2 (1.5	31 (23.1)
Sanguanwit (2021) [28]	Direct-L	80	65 (17.2)	40 (50)	-	-	-	-	-	-
	Video-L	78	73 (12.9)	44 (57)	-	-	-	-	-	-
Sulser (2016) [29]	Direct-L	73	55 (80.1)	-	-	-	-	-	-	-
	Video-L	74	68 (87.7)	-	-	-	-	-	-	-
Sun (2020) [30]	Direct-L	88	49.4 (9.7)	34 (38.60	23.6 (5.7)	-	-	-	-	-
	Video-L	88	49.3 (7.8)	39 (44)	23.7 (2.75)	-	-	-	-	-
Yeatts (2013) [31]	Direct-L	303	43 (12.6)	244 (76.3)	-	14 (4.4)	-	12 (3.8)	-	-
	Video-L	320	42 (16.83)	216 (71.3)	-	10 (3.3)	-	6 (2.0)	-	-

### The first attempt’s success rate

**Fig. 3:**
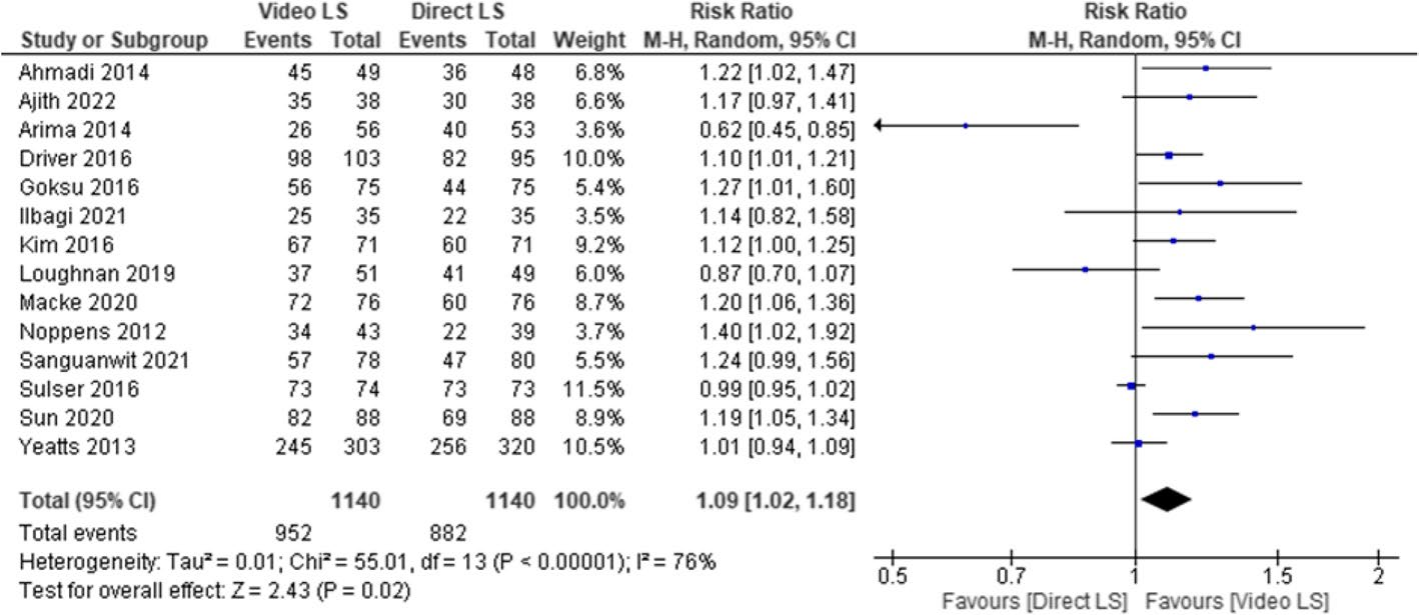
Forest plot comparing video laryngoscope and direct laryngoscope groups regarding the first-attempt success rate

The overall RR of the first-attempt success rate favored the VL group as it was associated with an increased chance of having success on the first attempt over DL group (*RR* = 1.09, 95% *CI* [1.02, 1.18], *P* = 0.02, Fig. 3). The pooled studies were not homogenous (*P* < 0.001; *I*^2^ = 76%). We ran the sensitivity analysis excluding one study in each scenario; however, the heterogeneity was not resolved.

### The first-attempt intubation time

The overall MD between the VL and DL groups showed that the VL group had a lower first-attempt intubation time than the DL group (*MD* =  − 6.92, 95% CI [− 12.86, − 0.99], *P* = 0.02, Figure S1). The pooled studies were not homogenous (*P* < 0.001; *I*^2^ = 89%). The heterogeneity was resolved by removing Sun et al. (2020) study (*P* = 0.85; *I*^2^ = 0%); however, the analysis still favored the VL group (*MD* =  − 3.22, 95% *CI* [− 4.20, − 2.25], *P* < 0.001, Figure S2).

### The overall intubation success rate

**Fig. 4:**
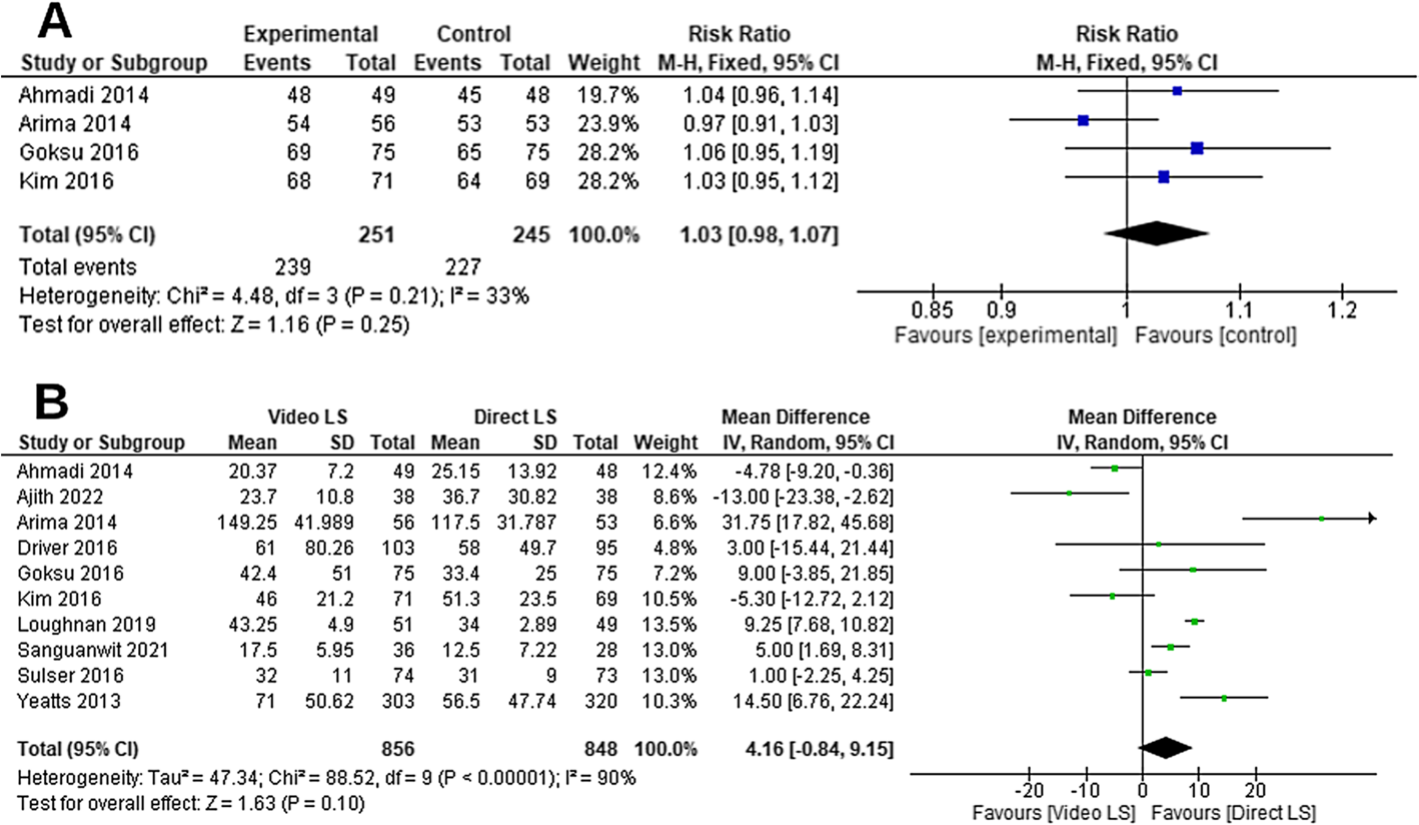
Forest plot comparing video laryngoscope and direct laryngoscope groups regarding **A** the overall intubation success rate and **B** the overall intubation time

The pooled RR of overall intubation success rate did not favor either VL or DL groups (*RR* = 1.03, 95% *CI* [0.98, 1.07], *P* = 0.25, Fig. 4A). The pooled studies were homogenous (*P* = 0.21; *I*^2^ = 33%).

### The overall intubation time

The overall MD between the VL and DL groups regarding the whole intubation time did not favor either of the two groups (*MD* = 4.16, 95% *CI* [− 0.84, 9.15], *P* = 0.1, Fig. 4B). The pooled studies were not homogenous (*P* < 0.001; *I*^2^ = 90%). We ran the sensitivity analysis excluding one study in each scenario; however, the heterogeneity was not resolved.

### The peri-intubation POGO score

The overall MD between VL and DL groups showed that the VL group had a better peri-intubation POGO scores than the DL group (*MD* = 24.91, 95% *CI* [11.18, 38.64], *P* < 0.001, Figure S3). The pooled studies were not homogenous (*P* = 0.003; *I*^2^ = 83%). The heterogeneity was resolved by removing Macke et al. (2020) study (*P* = 0.43; *I*^2^ = 0%); however, the analysis still favors the VL group (*MD* = 18.09, 95% *CI* [9.02, 27.16], *P* < 0.001, Figure S4).

### The IDS

The overall MD between the VL and DL groups showed that the VL group had a lower IDS than the direct laryngoscope group (*MD* =  − 0.62, 95% *CI* [− 0.86, 0.37], *P* < 0.001, Figure S5). The pooled studies were not homogenous (*P* = 0.02; I^2^ = 80%), and the sensitivity analysis was not applicable.

### The CL grading

#### Grade 1

The overall RR showed that the rate of grade 1 CL was significantly more in VL group than in the DL group (*RR* = 1.85, 95% *CI* [1.28, 2.66], *P* = 0.001, Figure S6). The pooled studies were homogenous (*P* = 0.17; *I*^2^ = 44%).

#### Grade 2A

The overall RR showed no significant difference between the two groups regarding the rate of grade 2A CL (*RR* = 0.85, 95% *CI* [0.56, 1.31], *P* = 0.46, Figure S7). The pooled studies were homogenous (*P* = 0.36; *I*^2^ = 2%).

#### Grade 2B

The overall RR showed that the rate of grade 2B CL was significantly lower in VL group than in DL group (*RR* = 0.49, 95% *CI* [0.25, 0.97], *P* = 0.04, Figure S8). The pooled studies were homogenous (*P* = 0.72; *I*^2^ = 0%).

#### Grade 3

The overall RR showed no significant difference between the two groups regarding the rate of grade 3 CL (*RR* = 0.53, 95% *CI* [0.24, 1.17], *P* = 0.12, Figure S9). The pooled studies were homogenous (*P* = 0.85; *I*^2^ = 0%).

### The rates of peri-intubation complications

#### Desaturation (hypoxia)

**Fig. 5:**
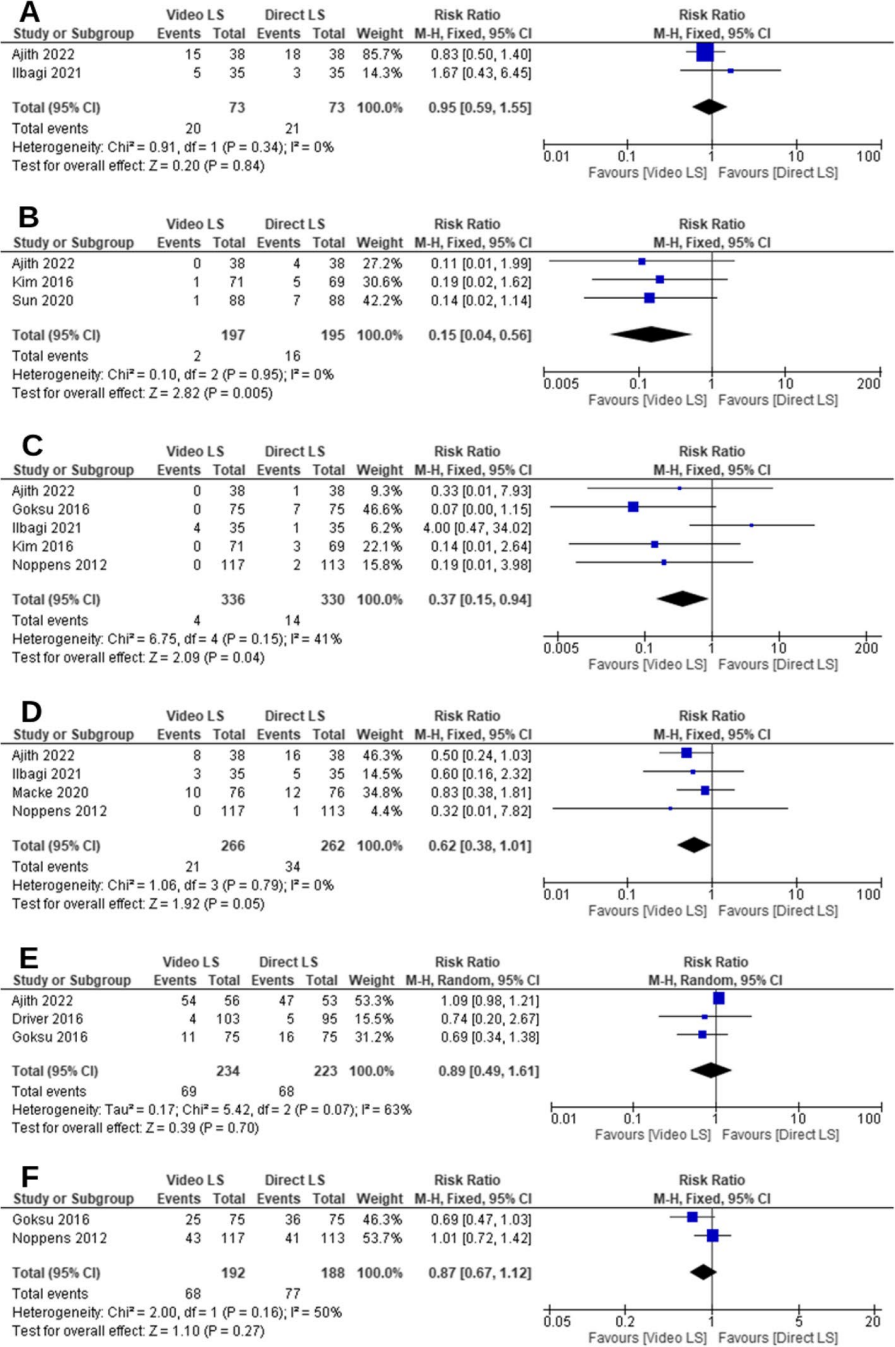
Forest plot comparing video laryngoscope and direct laryngoscope groups regarding the adverse events and complications. **A** The incidence of desaturation. **B** Upper airway injuries. **C** Esophageal intubation. **D** Aspiration. **E** Cardiac arrest. **F** The incidence of pO2 < 90

The pooled RR of the incidence of desaturation did not favor either of the two groups (*RR* = 0.95, 95% *CI* [0.59, 1.55], *P* = 0.84, Fig. 5A). The pooled studies were homogenous (*P* = 0.34; *I*^2^ = 0%).

#### Upper airway injuries (oropharynx or dental trauma)

The overall RR showed that VL group was significantly associated with a lower incidence of upper airway injuries compared to DL group (*RR* = 0.15, 95% *CI* [0.04, 0.56], *P* = 0.005, Fig. 5B). The pooled studies were homogenous (*P* = 0.95; *I*^2^ = 0%).

#### Esophageal intubation

The overall RR showed that VL group was significantly associated with a lower incidence of esophageal intubation compared to DL group (*RR* = 0.37, 95% *CI* [0.15, 0.94], *P* = 0.04, Fig. 5C). The pooled studies were homogenous (*P* = 0.15; *I*^2^ = 41%).

#### Aspiration

The incidence of aspiration was lower in VL group compared to DL group; however, it was not statistically significant (*RR* = 0.62, 95% *CI* [0.38, 1.01], *P* = 0.05, Fig. 5D). The pooled studies were homogenous (*P* = 0.79; *I*^2^ = 0%).

#### Cardiac arrest

The pooled RR of the incidence of cardiac arrest did not favor either of the two groups (*RR* = 0.89, 95% *CI* [0.49, 1.61], *P* = 0.70, Fig. 5E). The pooled studies were not homogenous (*P* = 0.07; *I*^2^ = 63%).

#### SpO2 < 90

The pooled RR of the incidence of SpO2 < 90 did not favor either of the two groups (*RR* = 0.87, 95% *CI* [0.67, 1.12], *P* = 0.27, Fig. 5F). The pooled studies were homogenous (*P* = 0.16; *I*^2^ = 50%).

### Publication bias assessment

By visual inspection of the funnel plot (Figure S10, Figure S11, Table S1), there was a possibility of publication bias as there was an asymmetrical distribution of the pooled studies in the funnel plot around the effect estimate. This asymmetrical distribution showed fewer studies in the direction of lower first-attempt success rates and a larger standard of error, which suggests that there might be unpublished studies with a small sample size that reported negative results (lower first-attempt success rates). Moreover, the Mazumdar rank correlation test (Kendall’s tau) and the regression test for funnel plot asymmetry showed no significant publication bias (*P* > 0.05). However, the fail-safe N test showed significant publication bias (*P* < 0.001).

## Discussion

Our findings indicate that while the overall intubation success rate was similar between the two groups, the video laryngoscope group demonstrated a higher first-attempt success rate, consistent with studies by Brown et al. (2020) [17], Okamoto et al. (2019) [32], and Sakles et al. (2012) [33]. Notably, in the context of trauma patients, Michailidou et al. (2015) [34] study showed a significantly higher success rate using VL compared to DL. Furthermore, recent propensity scores matched analysis focusing on trauma patients revealed that VL had twice the odds of first-pass success compared to DL (Trent et al., 2021) [35]. Many studies, although not specific to the emergency intubations, support the use of VL as the initial approach for patients with difficult airways [36–39]. These findings collectively suggest that VL may enhance first-attempt intubation success and improve visualization, particularly in challenging airways or emergencies where repeated attempts could pose risks or cause delays in airway management.

Although the overall intubation time did not significantly differ between the two groups, the video laryngoscope group demonstrated a shorter first-attempt intubation time, indicating the potential benefits of video technology in expediting and optimizing intubation procedures, thus enhancing patient comfort and procedural efficiency. Furthermore, trauma patients may oftentimes require many procedures alongside intubation. Optimizing intubation on the first attempt will allow for other necessary procedure to be performed even more efficiently.

VL eliminates the requirement for aligning the three airway axes and offers an enhanced view of the glottis with reduced force and manipulation of the cervical spine. Our study reveals that the video laryngoscope group achieved better POGO scores and lower intubation difficulty scores compared to the direct laryngoscope group. Additionally, we observed a higher rate of Cormack-Lehane grade 1 in the video laryngoscope group. These findings support the notion that VL enables improved visualization of the glottic structures, simplifying intubation and potentially reducing complications from challenging intubations. Okamoto et al. (2019) [32] study and multiple studies [40–43] in pediatric population beyond the emergency situations support this argument.

Our study shows that the video laryngoscope group exhibited lower rates of upper airway injuries, esophageal intubation, and aspiration compared to the direct laryngoscope group. These findings are consistent with studies by Okamoto et al. (2019) [32], Sakles et al. (2012) [33], and Sakles et al. (2015) [44], which also reported decreased complications with video laryngoscopy, particularly a reduced rate of esophageal intubation. Moreover, in the context of inhospital CPR, video laryngoscopy has been independently associated with successful endotracheal intubation on the first attempt and has shown a higher success rate compared to direct laryngoscopy (Lee et al., 2015) [45].

There have been almost 22 systematic reviews and meta-analyses published comparing video laryngoscopy to direct laryngoscopy, and they dealt with different aspects of endotracheal intubation. Downey et al. [46] reported findings of 21 meta-analyses on the comparison of video laryngoscopy versus direct laryngoscopy. A Cochrane review [47] also dealt with different forms of video laryngoscopy, i.e., Macintosh-style, hyper-angulated, and channeled laryngoscopy versus direct laryngoscopy for endotracheal intubation. These results are in agreement with our results. According to a previous meta-analysis by Bhattacharjee [48], they did not find any evidence of the superiority of video laryngoscopy over direct laryngoscopy in terms of the first intubation success rate, overall intubation success rate, and time to intubation. Another meta-analysis [49] also reported no statistically significant advantage of video laryngoscopy as compared to direct laryngoscopy for emergent intubation. Our results are not in agreement with these two previous meta-analyses in terms of the first intubation success rate and the first-attempt intubation time. The reason could be due to the difference in sample size as we included many new RCTs. It could also be due to low heterogeneity between studies. According to the majority of previous studies, video laryngoscopy increased the rate of success on the first intubation attempt and improved glottic view when assessed as Cormack-Lehane grades 3 and 4.

While previous systematic reviews and meta-analyses have compared video laryngoscopy to direct laryngoscopy in various contexts, our study differs by specifically focusing on the efficacy of VL versus DL for emergent intubations. By including only randomized controlled trials, we aimed to minimize biases associated with observational studies. Our findings align with the majority of previous studies, reporting increased first-attempt success rates and improved glottic visualization with VL [47, 48, 50–56].

Our meta-analysis has some limitations. There was considerable heterogeneity in some of the pooled analyses, and some of that heterogeneity was resolved by sensitivity analysis, and some did not resolve by the sensitivity analyses. This heterogeneity may be attributed to the use of different types of video laryngoscopes and variations in the populations studied. We did not subgroup the trials based on the type of video laryngoscope because the data reported was scarce. Additionally, reporting study data instead of patient-level data is another limitation.

Our study has various implications for healthcare and research. Our study helps clinicians to decide in favor of video laryngoscopy for emergent intubations as it is associated with a better first-attempt success rate, shorter first-attempt intubation time, and a lower rate of complications as compared to the direct laryngoscopy. More RCTs are needed that evaluate the effect of video laryngoscopy for emergent intubation. A closer look at the qualification level of the proceduralist is worth further investigating. More data on the pediatric population is also needed for more comprehensive determinations and recommendations.

## Conclusion

Although the overall intubation success rate and overall intubation time did not significantly differ when comparing video laryngoscopy to direct laryngoscopy, VL achieved a higher first-attempt success rate, shorter first-attempt intubation time, better glottic visualization, and lower rates of aspiration events, upper airway injuries, and esophageal intubation. These findings highlight the potential benefits of VL in challenging airways and emergent situations. Further randomized control trials for emergency intubations, tailored to specific patient populations, in particular the pediatric population, are needed before establishing VL as the overall preferred method for emergent intubations, where successful intubation on the first attempt is crucial to avoid complications and delays.

### Future consideration

Key areas that warrant investigation include determining operators’ level of comfort and training in using VL for emergent intubations and the need for focused training and proficiency, cost effective analysis, detailed evaluation of different types of VL, and clinical utility of VL in patient-specific population particularly among pediatric group.

### Supplementary Information

**Additional file 1: Figure S1.** Forest plot comparing video laryngoscope vs direct laryngoscope in first attempt intubation time. **Figure S2.** Forest plot comparing video laryngoscope vs direct laryngoscope in first attempt intubation time after the sensitivity analysis. **Figure S3.** Forest plot comparing video laryngoscope vs direct laryngoscope in peri-intubation POGO scores. **Figure S4.** Forest plot comparing video laryngoscope vs direct laryngoscope in peri-intubation percentage of glottis opening scores after the sensitivity analysis. **Figure S5.** Forest plot comparing video laryngoscope vs direct laryngoscope in intubation difficulty score. **Figure S6.** Forest plot comparing video laryngoscope vs direct laryngoscope in the rate of grade 1 Cormack-Lehane score. **Figure S7.** Forest plot comparing video laryngoscope vs direct laryngoscope in the rate of grade 2A Cormack-Lehane score. **Figure S8.** Forest plot comparing video laryngoscope vs direct laryngoscope in the rate of grade 2B Cormack-Lehane score. **Figure S9.** Forest plot comparing video laryngoscope vs direct laryngoscope in the rate of grade 3 Cormack-Lehane score. **Figure S10.** Funnel plot of the publication bias in first attempt success rates. **Figure S11.** Funnel plot of the publication bias in first attempt success rates.

**Additional file 2: Table S1.** PRISMA 2020 Checklist.

## Data Availability

The dataset supporting the conclusions of this article is included within the article and its Supplementary file 1.

## Abbreviations

RCT: Randomized controlled trial
CI: Confidence interval
RR: Risk ratio
MD: Mean difference
POGO: Percentage of glottis opening
IDS: Intubation difficulty score
CL: Cormack-Lehane grading
PRISMA: Preferred Reporting Items for Systematic Reviews and Meta-Analyses
VL: Video laryngoscopy
DL: Direct laryngoscopy
CPR: Cardiopulmonary resuscitation
PICO: Participants, interventions, comparison, and outcomes
ROB2: Cochrane risk-of-bias assessment tool 2
AE: Adverse event
PR: Prevalence ratio
OR: Odds ratio
ETT: Endotracheal tube
PEEP: Positive end-expiratory pressure
PaO2: Arterial oxygen partial pressure
FiO2: Fraction of inspired oxygen
TBI: Traumatic brain injury
ICP: Intracranial pressure
SOFA: Sequential Organ Failure Assessment
APACHE: Acute Physiology and Chronic Health Evaluation
PPV: Positive predictive value
NPV: Negative predictive value
