# Supplementary material for: Video laryngoscopy versus direct laryngoscopy in achieving successful emergency endotracheal intubations: a systematic review and meta-analysis of randomized controlled trials
Source: Syst Rev. 2024 Mar 12;13:85. doi: 10.1186/s13643-024-02500-9 (PMC10935931; doi:10.1186/s13643-024-02500-9)
Supplement: Supplementary file 1 — Additional file 1: Figure S1. Forest plot comparing video laryngoscope vs direct laryngoscope in first attempt intubation time. Figure S2. Forest plot comparing video laryngoscope vs direct laryngoscope in first attempt intubation time after the sensitivity analysis. Figure S3. Forest plot comparing video laryngoscope vs direct laryngoscope in peri-intubation POGO scores. Figure S4. Forest plot comparing video laryngoscope vs direct laryngoscope in peri-intubation percentage of glottis opening scores after the sensitivity analysis. Figure S5. Forest plot comparing video laryngoscope vs direct laryngoscope in intubation difficulty score. Figure S6. Forest plot comparing video laryngoscope vs direct laryngoscope in the rate of grade 1 Cormack-Lehane score. Figure S7. Forest plot comparing video laryngoscope vs direct laryngoscope in the rate of grade 2A Cormack-Lehane score. Figure S8. Forest plot comparing video laryngoscope vs direct laryngoscope in the rate of grade 2B Cormack-Lehane score. Figure S9. Forest plot comparing video laryngoscope vs direct laryngoscope in the rate of grade 3 Cormack-Lehane score. Figure S10. Funnel plot of the publication bias in first attempt success rates. Figure S11. Funnel plot of the publication bias in first attempt success rates. [file 13643_2024_2500_MOESM1_ESM.docx]

Figure S1: Forest plot comparing video laryngoscope vs direct laryngoscope in first attempt intubation time:


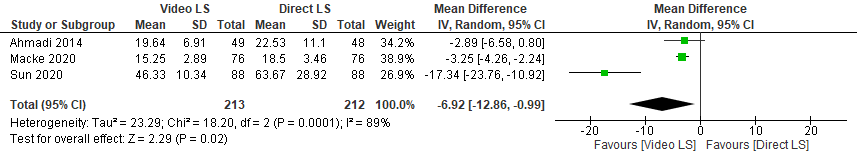


Figure S2: Forest plot comparing video laryngoscope vs direct laryngoscope in first attempt intubation time after the sensitivity analysis:


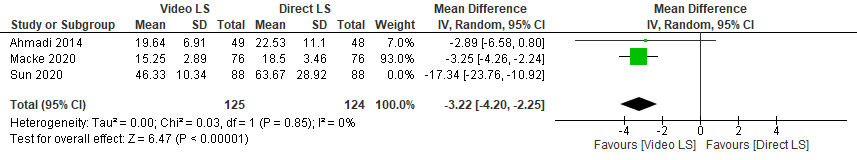


Figure S3: Forest plot comparing video laryngoscope vs direct laryngoscope in peri-intubation POGO scores:


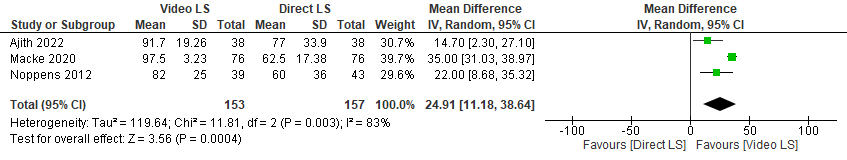


Figure S4: Forest plot comparing video laryngoscope vs direct laryngoscope in peri-intubation percentage of glottis opening scores after the sensitivity analysis:


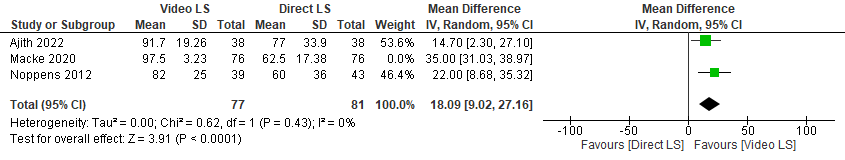


Figure S5: Forest plot comparing video laryngoscope vs direct laryngoscope in intubation difficulty score:


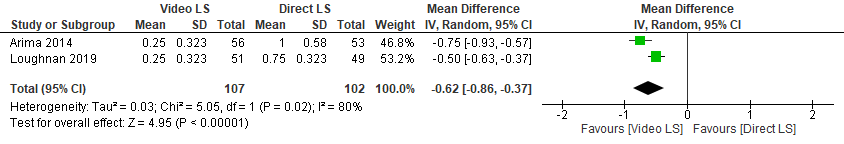


Figure S6: Forest plot comparing video laryngoscope vs direct laryngoscope in the rate of grade 1 Cormack-Lehane score:


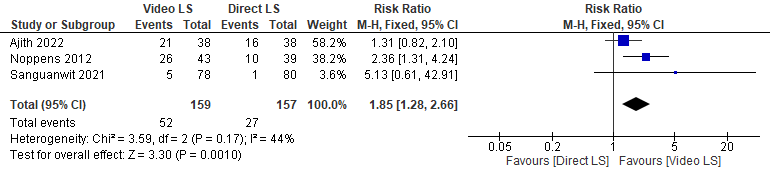


Figure S7: Forest plot comparing video laryngoscope vs direct laryngoscope in the rate of grade 2A Cormack-Lehane score:


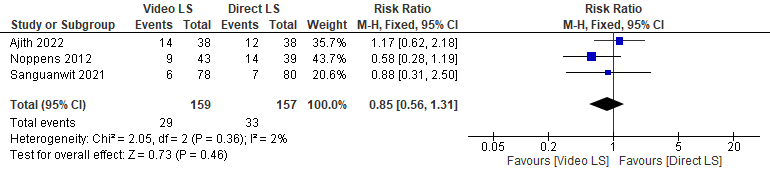


Figure S8: Forest plot comparing video laryngoscope vs direct laryngoscope in the rate of grade 2B Cormack-Lehane score:


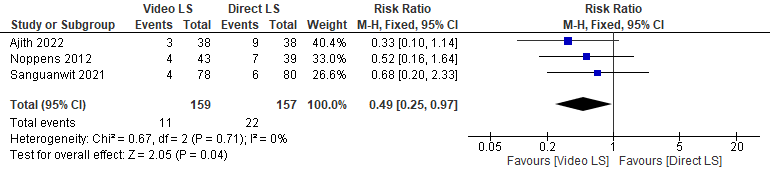


Figure S9: Forest plot comparing video laryngoscope vs direct laryngoscope in the rate of grade 3 Cormack-Lehane score:


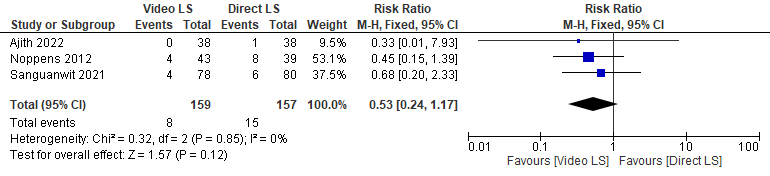


Figure S10: Funnel plot of the publication bias in first attempt success rates:


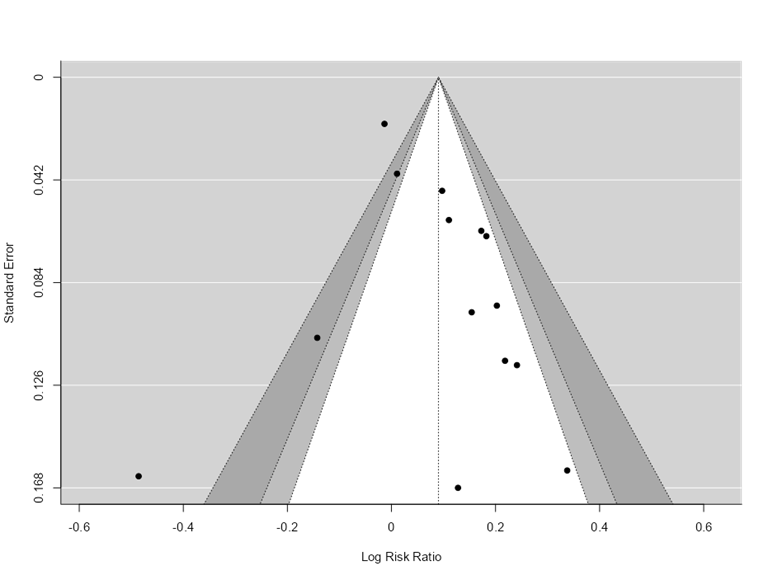


Figure S11: Funnel plot of the publication bias in first attempt success rates:


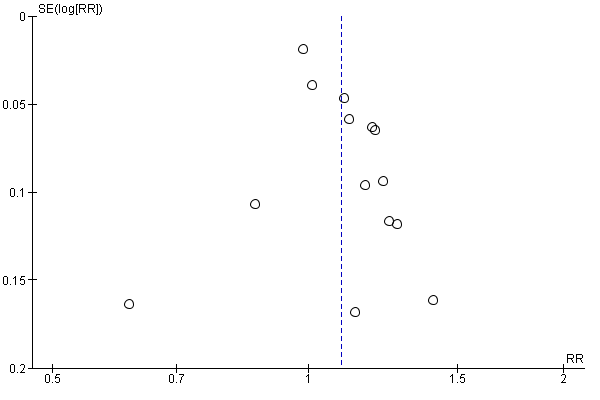


Table S1: The publication bias tests’ results

| Test name | Test statistics | P-value |
| --- | --- | --- |
| Fail-Safe N Analysis (File Drawer Analysis) | 71.000 | **< 0.001** |
| Rank Correlation Test for Funnel Plot Asymmetry | Kendall's Tau= 0.209 | 0.331 |
| Regression Test for Funnel Plot Asymmetry | Z= 0.327 | 0.744 |
